# Efficacy of flap repair for anal fistula: study protocol for a systematic review of randomized controlled trial

**DOI:** 10.1097/MD.0000000000016856

**Published:** 2019-08-16

**Authors:** Hao Zhang, Tao Xu, Xiao-dong Zhang

**Affiliations:** a Department of Plastic Burn and Cosmetic Center; b Department of Medical Imaging, First Affiliated Hospital of Jiamusi University, Jiamusi, China.

**Keywords:** anal fistulas, efficacy, flap repair, randomized controlled trial, safety

## Abstract

**Background:**

Clinical trials have reported that flap repair (FR) can treat anal fistula (AF) effectively. However, no study systematically investigated its efficacy and safety for patients with AF. This study will systematically assess its efficacy and safety of AF.

**Methods:**

We will retrieve MEDLINE, EMBASE, Cochrane Library, Elsevier, Springer, Web of Science, Scopus, Chinese Biomedical Literature Database, China National Knowledge Infrastructure, VIP Information, and Wanfang Data from their inceptions to May 1, 2019 without any language limitations. The primary outcome is fistula cure rate. The secondary outcomes consist of fistula recurrence rate, fecal continence, quality of life, and complications. RevMan 5.3 software will be used for methodological quality assessment, data synthesis, subgroup analysis and sensitivity analysis.

**Results:**

The results of this study will summarize a high-quality synthesis of current evidence for the treatment of FR for patients with AF.

**Conclusion:**

The findings of this proposed study will provide evidence for judging whether FR is an effective and safety intervention for AF or not.

PROSPERO registration number: PROSPERO CRD42019135507.

## 1 Introduction

Anal fistula (AF) is a very common anal disorder, which is the main etiology of perianal abscesses and suppurations.^[1,2]^ It mainly manifests as a variety of symptoms, such as pain, fecal incontinence, impaired quality of life and work incapacity.^[3,4,5,6]^ It has been estimated that more than 1:10,000 individuals can affect such disorder.^[7]^ Its prevalence rate in men to women is 1.23: 0.56 per 1000 population with an average age of 40 years.^[8,9,10]^

Several managements are responsible for the treatment of AF.^[11,12,13,14,15,16,17,18,19,20]^ Of them, flap repair (FR) is reported for treating AF.^[11,17]^ However, no study has been systematically assessed the efficacy and safety of FR for the treatment of AF. Thus, in this study, we will evaluate the efficacy and safety of FR for patients with AF.^[11,17]^

## 2 Methods and materials

### 2.1 Inclusion and exclusion criteria for study selection

#### 2.1.1 Types of studies

Only randomized controlled trials (RCTs) regarding the efficacy and safety of FR for patients with AF will be included. Non-clinical studies, non-controlled trials, and non-RCTs will be excluded.

#### 2.1.2 Types of participants

All patients of clinically diagnosed with AF will be included without any restrictions of gender and age.

#### 2.1.3 Types of interventions

The patients in the experimental group receive FR. The patients in the control group receive any treatments, except FR.

#### 2.1.4 Types of outcomes

The primary outcome is Fistula cure rate. The cure was defined as complete wound healing with absence of symptoms.

The secondary outcomes consist of fistula recurrence rate; fecal continence, as measured by Rockwood Fecal Incontinence Severity Index or other relevant scales; quality of life, as measured by Global Quality of Life Scale or other associated scales; and complications.

### 2.2 Literature search strategy

Relevant RCTs of FR for patients with AF from the databases including the MEDLINE, EMBASE, Cochrane Library, Elsevier, Springer, Web of Science, Scopus, Chinese Biomedical Literature Database, China National Knowledge Infrastructure, VIP Information, and Wanfang Data from their inceptions to May 1, 2019 without any language limitations. The strategy for searching MEDLINE is presented as an example in Table 1. The similar modified strategy will be used to other electronic databases.

**Table 1:**
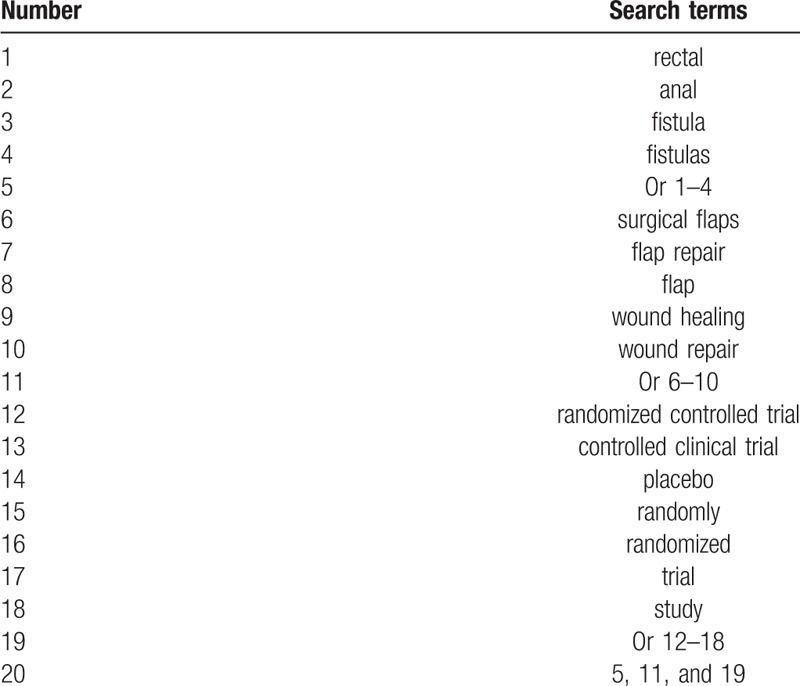
Search strategy for MEDLINE.

In addition, we will also search grey literatures, including conference proceedings, dissertations, and reference lists of all eligible RCTs to avoid missing any potential studies.

### 2.3 Data collection

#### 2.3.1 Study selection

Two reviewers will independently read all the titles and abstracts of all searched literature. Then, all duplicated and irrelevant studies will be excluded based on the eligibility criteria. After that, the full text of remaining literatures will be read to judge whether those studies meet the final eligible criteria. A third reviewer will be invited to solve any disagreements, raised between two reviewers regarding any issues of study selection. The whole process of study selection will be presented in the flowchart of Preferred Reporting Items for Systematic Reviews and Meta-Analyses with specific reason for each excluded study at different stages.

#### 2.3.2 Data extraction and management

Two reviewers will independently extract data from all eligible literatures through prediluted data extraction sheet. Any divergences will be settled down by discussion with a third reviewer invited. The extracted information comprises of

1) General information: title, first author, year of publication, inclusion and exclusion criteria, etc;
2) Methods: sample size, randomization, concealment, blinding, etc;
3) Treatments: name of different interventions, control, dosage, frequency, etc;
4) Outcomes: Primary, secondary, and safety outcomes, etc.

#### 2.3.3 Missing data dealing with

If any insufficient information or missing data will occur, we will contact primary authors to obtain those data. If we cannot receive that information, we will only analyze the available data, and will discuss the potential impacts of missing information.

#### 2.3.4 Methodological quality assessment for eligible studies

Cochrane Risk of Bias Tool will be utilized for assessing the methodological quality of all eligible studies. It consists of 7 domains, and each aspect is further divided into 3 degrees: low, unclear, and high risk of bias. Two reviewers will independently assess the methodological quality for each eligible study. Any disagreements regarding the methodological quality assessment between two reviewers will be solved by a third reviewer through discussion.

### 2.4 Statistical analysis

#### 2.4.1 Treatment effect measurements

Measurement data will be expressed as mean difference or standardized mean difference with 95% confidence intervals. Enumeration data will be expressed as relative risk or odds ratio with 95% confidence intervals.

#### 2.4.2 Heterogeneity identification

The *I* ^*2*^ test is used for identified heterogeneity among eligible studies. *I* ^*2*^ ≤ 50% means low heterogeneity among included studies, while *I* ^*2*^ > 50% means significant heterogeneity. Then, subgroup analysis or sensitivity analysis will be carried out to identify the source of heterogeneity.

#### 2.4.3 Data synthesis

RevMan 5.3 software will be used for meta-analysis performance. If there is small heterogeneity, a fixed-effects model will be utilized for analysis. If there is substantial heterogeneity among eligible studies, a random-effects model will be applied, and subgroup analysis will be carried out. If substantial heterogeneity is still identified after subgroup analysis, we will not pool data, and will report outcome results as narrative descriptions instead of meta-analysis performance.

#### 2.4.4 Subgroup analysis

Subgroup analysis will be employed in according to the different study characteristics, treatments, controls, and outcome measurements.

#### 2.4.5 Sensitivity analysis

Sensitivity analysis will be carried out to check robustness of pooled data by removing low quality studies.

#### 2.4.6 Publication bias

In this study, we will perform funnel plot,^[21]^ and Egger regression^[22]^ to identify any reporting bias if more than 10 studies are included.

## 3 Discussion

To the best of our knowledge, this is the first study to focus on assessing the efficacy and safety of FR for the treatment of patients with AF. The results of this study will determine if FR is a superior modality for patients with AF. Moreover, we also believe that this study may provide helpful evidence for clinical recommendations, which may benefit for either patients or clinical practice, as well as help clinicians to make the best choice for such patients.

## Author contributions

**Conceptualization:** Hao Zhang, Tao Xu, Xiao-dong Zhang.

**Data curation:** Hao Zhang, Tao Xu, Xiao-dong Zhang.

**Formal analysis:** Tao Xu, Xiao-dong Zhang.

**Funding acquisition:** Hao Zhang.

**Investigation:** Hao Zhang.

**Methodology:** Tao Xu, Xiao-dong Zhang.

**Project administration:** Hao Zhang.

**Resources:** Tao Xu, Xiao-dong Zhang.

**Software:** Tao Xu, Xiao-dong Zhang.

**Supervision:** Hao Zhang.

**Validation:** Hao Zhang, Tao Xu, Xiao-dong Zhang.

**Visualization:** Hao Zhang.

**Writing – original draft:** Hao Zhang, Tao Xu, Xiao-dong Zhang.

**Writing – review & editing:** Hao Zhang, Tao Xu, Xiao-dong Zhang.

## References

[1] Stupart D Goldberg P Levy A Tuberculous anal fistulas--prevalence and clinical features in an endemic area. S Afr J Surg 2009;47:116–8. 20141068

[2] Lee CL Lu J Lim TZ Long-term outcome following advancement flaps for high anal fistulas in an Asian population: a single institution's experience. Int J Colorectal Dis 2015;30:409–12. 25575433 10.1007/s00384-014-2100-y

[3] Lilius HG Etiology and pathogenesis of anal fistulas. Duodecim 1969;85:417–22. 4895028

[4] Sainio P Husa A Management of anal fistulas. Duodecim 1987;103:348–53. 3311700

[5] Senéjoux A Conventional surgical treatment of anal fistulas. Ann Chir 2004;129:611–5. 15581825 10.1016/j.anchir.2004.10.007

[6] Schwandner O Quality indicators in the treatment of anal fistulas. Chirurg 2019;90:270–8. 30683947 10.1007/s00104-019-0794-7

[7] Tabry H Farrands PA Update on anal fistulae: surgical perspectives for the gastroenterologist. Can J Gastroenterol 2011;25:675–80. 22175058 10.1155/2011/931316 PMC3266159

[8] Leng Q Jin HY Anal fistula plug vs mucosa advancement flap in complex fistula-in-ano: a meta-analysis. World J Gastrointest Surg 2012;4:256–61. 23494149 10.4240/wjgs.v4.i11.256 PMC3596507

[9] Parks AG Gordon PH Hardcastle JD A classification of fistula-in-ano. Br J Surg 1976;63:1–2. 1267867 10.1002/bjs.1800630102

[10] Simpson JA Banerjea A Scholefield JH Management of anal fistula. BMJ 2012;345:e6705. 23069597 10.1136/bmj.e6705

[11] Dubois A Carrier G Pereira B Therapeutic management of complex anal fistulas by installing a nitinol closure clip: study protocol of a multicentric randomised controlled trial--FISCLOSE. BMJ Open. 2015; 5(12):e009884. 10.1136/bmjopen-2015-009884 PMC4691740 26674505

[12] Stamos MJ Snyder M Robb BW Prospective multicenter study of a synthetic bioabsorbable anal fistula plug to treat cryptoglandular transsphincteric anal fistulas. Dis Colon Rectum 2015;58:344–51. 25664714 10.1097/DCR.0000000000000288

[13] Sözener U Gedik E Kessaf Aslar A Does adjuvant antibiotic treatment after drainage of anorectal abscess prevent development of anal fistulas? A randomized, placebo-controlled, double-blind, multicenter study. Dis Colon Rectum 2011;54:923–9. 21730779 10.1097/DCR.0b013e31821cc1f9

[14] Schwandner T Roblick MH Kierer W Surgical treatment of complex anal fistulas with the anal fistula plug: a prospective, multicenter study. Dis Colon Rectum 2009;52:1578–83. 19690485 10.1007/DCR.0b013e3181a8fbb7

[15] Fukuda Y Takazoe M Sugita A Oral spherical adsorptive carbon for the treatment of intractable anal fistulas in Crohn's disease: a multicenter, randomized, double-blind, placebo-controlled trial. Am J Gastroenterol 2008;103:1721–9. 18616656 10.1111/j.1572-0241.2008.01860.x

[16] Witte ME Klaase JM Gerritsen JJ Fibrin glue treatment for simple and complex anal fistulas. Hepatogastroenterology 2007;54:1071–3. 17629041

[17] Ellis CN Clark S Fibrin glue as an adjunct to flap repair of anal fistulas: a randomized, controlled study. Dis Colon Rectum 2006;49:1736–40. 17053867 10.1007/s10350-006-0718-8

[18] Vitton V Gasmi M Barthet M Long-term healing of Crohn's anal fistulas with fibrin glue injection. Aliment Pharmacol Ther 2005;21:1453–7. 15948812 10.1111/j.1365-2036.2005.02456.x

[19] van der Hagen SJ Baeten CG Soeters PB Anti-TNF-alpha (infliximab) used as induction treatment in case of active proctitis in a multistep strategy followed by definitive surgery of complex anal fistulas in Crohn's disease: a preliminary report. Dis Colon Rectum 2005;48:758–67. 15750797 10.1007/s10350-004-0828-0

[20] Faucheron JL Saint-Marc O Guibert L Long-term seton drainage for high anal fistulas in Crohn's disease--a sphincter-saving operation? Dis Colon Rectum 1996;39:208–11. 8620789 10.1007/BF02068077

[21] Sutton AJ Duval SJ Tweedie RL Empirical assessment of effect of publication bias on meta-analyses. BMJ 2000;320:1574–7. 10845965 10.1136/bmj.320.7249.1574 PMC27401

[22] Egger M Davey Smith G Schneider M Bias in meta-analysis detected by a simple, graphical test. BMJ 1997;315:629–34. 9310563 10.1136/bmj.315.7109.629 PMC2127453

